# Sensory mutations in *Drosophila melanogaster* influence associational effects between resources during oviposition

**DOI:** 10.1038/s41598-017-09728-7

**Published:** 2017-08-24

**Authors:** Thomas A. Verschut, Mikael A. Carlsson, Peter Anderson, Peter A. Hambäck

**Affiliations:** 1 Department of Ecology, Environment and Plant Sciences, Stockholm University, 106 91 Stockholm, Sweden; 2 Department of Zoology, Stockholm University, 106 91 Stockholm, Sweden; 3 Department of Plant Protection Biology, Swedish University of Agricultural Sciences, Box 102, 230 53 Alnarp, Sweden

## Abstract

Neighboring resources can affect insect oviposition behavior when the complexity of sensory information obscures information about host resource availability in heterogeneous resource patches. These effects are referred to as associational effects and are hypothesized to occur through constraints in the sensory processing of the insect during host search, resulting into suboptimal resource use. Because the possibilities to study these constraints on naturally occurring animals are limited, we instead used sensory mutants of *Drosophila melanogaster* to determine the importance of sensory information in the occurrence of associational effects. We found that oviposition was mainly governed by non-volatile chemical cues and less by volatile cues. Moreover, the loss of gustatory sensilla resulted in random resource selection and eliminated associational effects. In conclusion, our study shows that associational effects do not necessarily depend on constraints in the sensory evaluation of resource quality, but may instead be a direct consequence of distinctive selection behavior between different resources at small scales.

## Introduction

Insect oviposition involves several behavioral steps in which females use multiple sensory modalities to evaluate cues indicating the quality of the available resources for progeny development^1–4^. While numerous studies have shown that the degree to which insects distribute their eggs among hosts can have profound consequences for their reproductive fitness^5–7^, it has remained difficult to determine how resource heterogeneity affects the selection behavior of insects among alternative hosts. One way to disentangle the consequences of resource heterogeneity on host selection is by looking at associational effects, which allows you to determine whether the likelihood that an insect selects a particular host resource changes due to the presence of neighboring resources^8–10^. Associational effects have been hypothesized to occur due to limitations in the sensory physiology of insects, which constrain their ability to evaluate resource quality at different levels of host search^11–13^. It is, for example, possible that the resolution of sensory information used to locate a resource patch will not be sufficient to distinguish between the qualities of the individual resources within the patch^14, 15^. Consequently, constraints in resource evaluation may cause female insects to under- or overestimate the actual profitability of a patch and change the likelihood of selecting a particular oviposition resource in low and high quality neighborhoods^11–13^.

**Figure 1:**
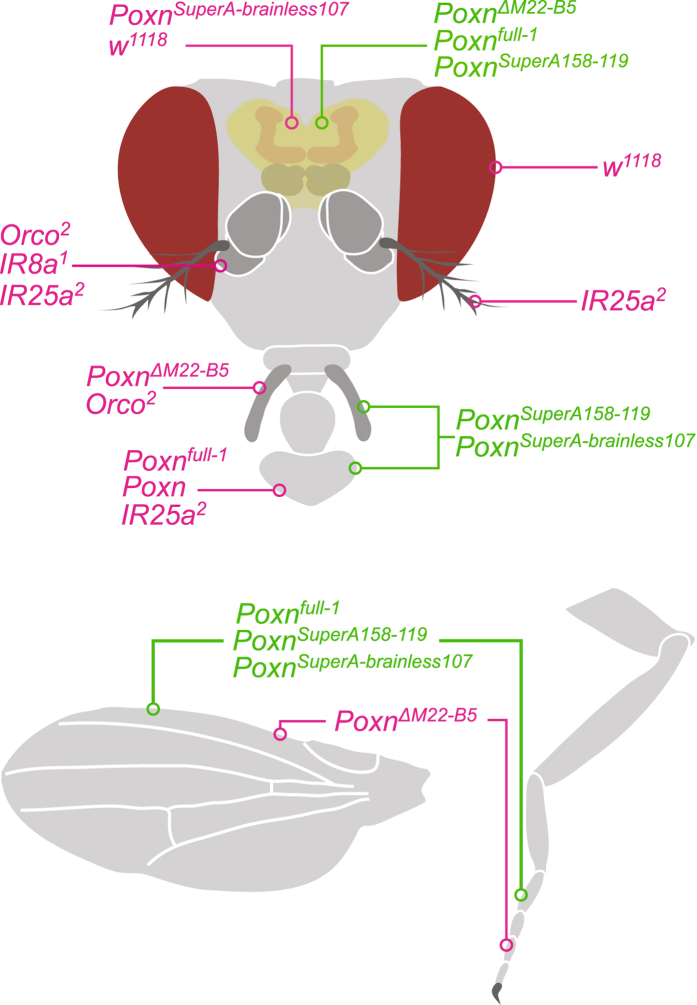
Representation of the sensory deficiencies in the mutant strains used in our oviposition experiment. The strains in which mutations caused deficiencies in sensory modalities are indicated with magenta, and the strains in which the functioning of sensory modalities were restored are indicated with green. Additional background information and stock numbers can be found in Supplementary Table S1.

The ability to evaluate resource quality is presumably lower from a distance, and previous studies have therefore mainly examined long-range resource selection as a mechanism for associational effect^8–10^. In this study, we instead examined the possibility that short-range selection may also cause associational effects. Moreover, we also took advantage of the ongoing development of sensory mutants of *Drosophila melanogaster* Meigen (Diptera: Drosophilidae), to investigate which sensory modalities could be involved in the occurrence of associational effects during short-range resource selection. Many of the molecular studies on sensory mutations have been pivotal in understanding the physiology and evolution of the insect sensory system^16–19^, but can nowadays also help to gain insight into how sensory information affects insect search behavior^20–22^. For this reason, we used *D. melanogaster* as a model organism to study how sensory information can generate associational effects by comparing the resource selection behavior of wild type flies with that of strains with olfactory and gustatory deficiencies (Fig. 1; Supplementary Table S1), using an oviposition assay in which we manipulated the frequency of two alternative oviposition resources.

**Figure 2:**
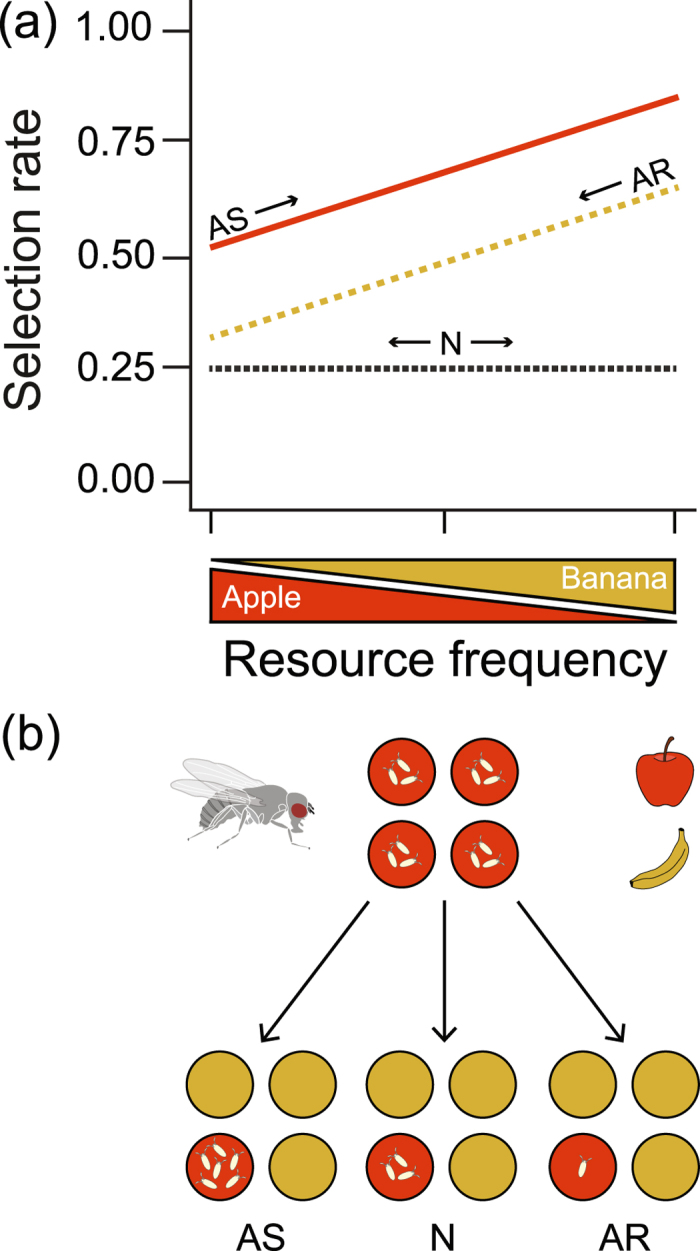
A conceptual framework for the occurrence of associational effects in the oviposition assay. Associational effects occur when the oviposition rate on a resource decreases or increases in the presence of neighboring resources. In (**a**) apple (red - solid line) experiences associational susceptibility (AS) as the relative selection rate increases with the increasing frequency of banana in the patch. Banana (yellow - dashed line) experiences associational resistance (AR) as the selection rate on banana decreases with the decreasing frequency of banana in the patch. Although the lines have been drawn parallel to each other for visual simplicity, stronger increases in the oviposition rates on one resource type in comparison to the other resource type can result in non-parallel lines, while still representing the same combination of associational effects. When the frequency of neighboring resources has a neutral effect on the oviposition rate (N - grey dashed line) no associational effects occur for that resource. In (**b**) the different associational effects are illustrated by the number of eggs on apple oviposition resources. When comparing the number of eggs on individual apple resources in pure patches with that in mixed patches, the oviposition rate on apple can either decrease (AR), stay the same (N), or increase (AS). Banana can simultaneously experience associational effects but these effects are not illustrated in this example.

With our experimental setup, we studied the behavioral process involved in the short-range evaluation of oviposition resources separately from larger scale behavioral processes preceding the oviposition choice. Consequently, we eliminated the possibility that constraints in the sensory system reduce the ability to evaluate resource quality at the level of a patch or individual resource, and only allowed associational effects to be generated through resource selection at very small scales (for further explanation see Fig. 2). Our results revealed that the occurrence of associational effects does not depend on sensory constraints between the level of a patch and individual resource, but in contrast, may also arise whenever insects distinctively select between two resources at very small scales. Our study also shows that in the case of *D. melanogaster*, this selection is mainly governed by the gustatory sensory system and less by the olfactory sensory system.

### Oviposition behavior of wild type *Drosophila*

**Figure 3:**
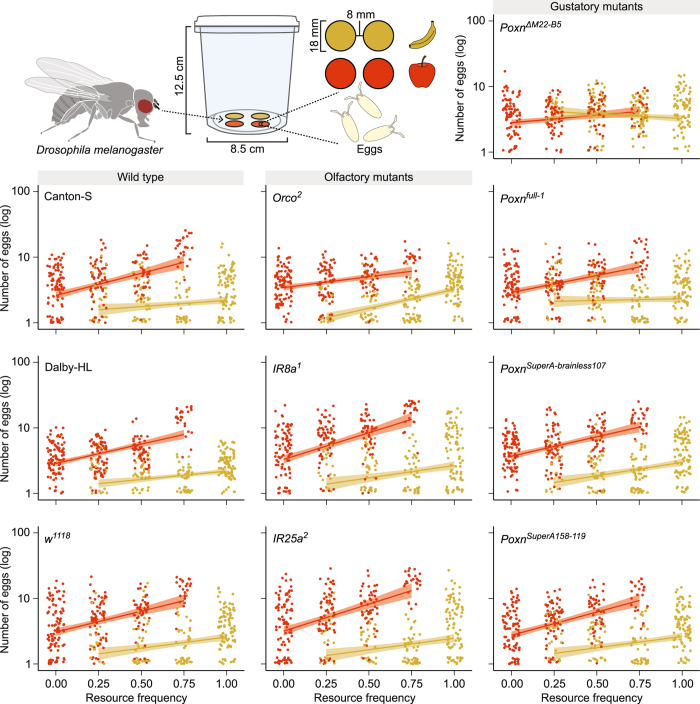
Oviposition behavior of wild type flies and sensory mutants. A graphic representation of the oviposition assay is given at the top of the figure. Each point in the graph represents the number of eggs laid by an individual fruit fly female on either an apple (red) or banana (yellow) oviposition disc (log scale). The x-axis represents the increasing frequency of banana oviposition discs in the patch. In the opposite direction, the frequency of banana also provides the frequency of apple in the patch (i.e. 1.00 minus the frequency of banana). The predicted linear regression lines for oviposition on either resource types are illustrated with their 95% confidence interval. The points representing egg distribution are jittered vertically and horizontally for visualization purposes.

Previous studies on long-range resource selection through olfactory cues showed that when two resources co-occur, the attraction rate of *D. melanogaster* increases towards preferred resources, and decreases towards less preferred resources, resulting in associational susceptibility (AS) for the former and associational resistance (AR) for the latter^23, 24^. This combination of AS and AR is generally expected to occur when insects already select resources though long-range olfactory information before entering a resource patch^25^. However, it is still unknown whether resource selection within a patch, which often depends on sensory information only perceived at much shorter ranges^14, 15^, generates similar patterns of associational effects between preferred and less preferred resources. To examine this possibility, we performed oviposition experiments in which individual wild type *D. melanogaster* females (Supplementary Table S1; Canton-S, Dalby-HL and *w* ^1118^) were offered four oviposition discs either containing apple or banana fruit pulp (i.e. oviposition discs - Fig. 3). We tested the oviposition behavior across a wide range of resource frequencies, and found that wild type fruit flies strongly preferred to oviposit on apple discs rather than on banana discs (GLM: *χ* ^2^_1,1489_ = 61.64, *P* < 0.001), and that the overall oviposition rate is positively affected by the increasing frequency of banana in the patch (GLM: *χ* ^2^_1,1489_ = 78.84, *P* < 0.001; Fig. 3). Moreover, we also found that the oviposition rates were affected by an interaction between resource type and resource frequency (GLM: *χ* ^2^_1,1489_ = 11.35, *P* < 0.001), which indicates that the relative oviposition rate on apple changed more in the presence of banana than the oviposition rate on banana changed in the presence of apple (Fig. 3). As these effects did not vary between the three strains (GLM: *χ* ^2^_2,1489_ = 3.91, *P* = 0.14; Supplementary Tables S2 and S3), we can assume that the observed oviposition patterns are not specific to a particular wild type strain.

The observed oviposition patterns would translate into associational effects when the oviposition rate on either resource type differs in resource mixtures from the oviposition rate in environments with only one resource type^23, 24^. Evidently, the observed patterns translated to AS for apple, because the oviposition rate on apple increased with the decreasing frequency of apple in the patch, and AR for banana as the oviposition rate on banana decreased with the decreasing frequency of banana. Because our oviposition assay effectively studies small-scale decisions made during oviposition selection within the patch, our results suggest that associational effects do not necessarily rely on constraints in the ability to interpret sensory information at different levels of search behavior^11–13^. In contrast, our results suggest that associational effects can also occur when insects distinctively select between two resources at a very small spatial scale. We next investigated which sources of short-range sensory information were used by the fruit flies to select for either of the two oviposition resources within our oviposition assay. We hypothesized that reducing the sensory capabilities of the fruit flies might lower their abilities to distinguish between the two oviposition substrates and alter the associational effects between apple and banana. To validate this hypothesis, we made use of olfactory and gustatory mutants to determine if deficiencies in these sensory modalities influence the oviposition choice of fruit flies (Fig. 1; Supplementary Table S1), and thereby affect the patterns of the associational effects we found in the experiments with the wild type flies.

### Oviposition behavior of olfactory mutants

We compared the oviposition behavior of three olfactory mutant strains (*Orco* ^2^, *IR8a* ^1^ and *IR25a* ^2^; Fig. 1; Supplementary Table S1) with that of the wild type *w* ^1118^ strain to determine if deficiencies in the olfactory system affects resource selection and could underlie the associational effects observed for the wild type fruit flies. Insect olfactory receptors function as a heteromeric complex formed by ligand-binding odorant receptors (ORs) and a chaperon co-receptor (Orco) that complements the signal transduction pathway^26–28^. In addition to the ORs, there is a second family of odorant receptors, the ionotropic glutamate-like receptors (IRs), of which several are dependent on either of the two broadly expressed co-receptors IR8a and IR25a^29, 30^. Due to the ubiquitous expression of Orco in all sensory neurons housing ORs^28, 31^, *Orco* ^2^ mutants can be assumed to have larger sensory deficiencies than *IR8a* ^1^ and *IR25a* ^2^ mutant flies, which respectively only lack the dedicated ionotropic co-receptors for the reception of certain carboxylic acids and amines^29, 30, 32^. The reason for comparing the behavior of the mutant strains with that of the *w* ^1118^ strain is that all sensory mutants contain the *white (w)* mutation in their genetic background (Supplementary Table S1), making *w* ^1118^ the preferred strain to represent wild type oviposition behavior in pairwise comparisons rather than the two other wild type strains.

These comparisons revealed no differences between the oviposition behavior of the *IR8a* ^1^ and *IR25a* ^2^ flies and that of the wild type *w* ^1118^ flies (Supplementary Table S4). However, when comparing *w* ^1118^ and *Orco* ^2^ flies we found interactions between resource frequency, resource type and strain affecting the egg distribution patterns (GLM: *χ* ^2^_1,990_ = 16.45, *P* < 0.001; Supplementary Table S4). When comparing the three olfactory mutants, we similarly found a three-way interaction between resource frequency, resource type and strain (GLM: *χ* ^2^_1,1487_ = 8.81, *P* = 0.01; Supplementary Table S5). These three-way interactions resulted from the presence of an interaction between resource frequency and resource type for the *Orco* ^2^ flies (GLM: *χ* ^2^_1,495_ = 23.77, *P* < 0.001) and the absence of such an interaction for both the *IR8a* ^1^ and *IR25a* ^2^ flies (Fig. 3; Supplementary Table S3; Table S5). More specifically, the behavior of the *Orco* ^2^ flies separates itself from the behavior of *w* ^1118^, *IR8a* ^1^ and *IR25a* ^2^ flies because the oviposition rate of the *Orco* ^2^ flies on apple increased much less with resource frequency (less strong AS) than the oviposition rate on banana (Fig. 3). Similar to the wild type flies, the oviposition rate of *Orco* ^2^ flies on apple still increased with the decreasing frequency of apple in the patch (AS). These results suggest that, although olfactory cues might be of importance in long-range resource selection^23, 24^, the odorants experienced by the flies in our small-scale oviposition assay are not the main source of short-range sensory information used by the fruit flies to distinguish between the two oviposition resources.

### Oviposition behavior of gustatory mutants

Because our experiments with the olfactory mutants showed that olfactory mediated selection does not explain the observed associational effects, we next examined the role of non-volatile chemical cues and gustatory receptors in generating these patterns. The most dominantly used receptors in evaluating non-volatile chemical cues are the gustatory receptors (GRs), but broadly tuned IRs and transient receptor potential channels (TRP) have also been found to be of importance^33^. The GRs are mainly housed in sensilla on the labellum, the anterior wing margins and the tarsi^34, 35^, but additional sensilla surrounding the ovipositor allow female flies to identify optimal nutritional conditions while selecting oviposition substrates^34, 36, 37^. We studied the behavior of *Pox-neuro* mutants (*Poxn*), with varying degrees of reduced functioning of the gustatory sensilla, to determine the degree to which non-volatile chemical cues are involved in generating associational effects. The *Poxn* mutation affects the development of the central and peripheral nervous system by turning all poly-innervated gustatory bristles into mono-innervated mechanosensory bristles, eliminating the direct-contact gustatory sensilla in the *Poxn* ^*ΔM22-B5*^ mutant flies^38–41^. We also tested *Poxn* ^*full-1*^ flies in which all gustatory sensilla except for those found on the labellum are restored. Finally, we tested the behavior of *Poxn* ^*SuperA-brainless107*^ in which all gustatory sensilla are restored but not the *Poxn* brain development, and *Poxn* ^*SuperA158-119*^ in which all gustatory sensilla and the *Poxn* brain development are restored (Fig. 1; Supplementary Table S1).

The comparisons between *w* ^1118^ and *Poxn* ^*ΔM22-B5*^, and between *w* ^1118^ and *Poxn* ^*full-1*^ mutant flies, showed effects of interactions between resource frequency and resource type, resource frequency and strain, and resource type and strain on the egg distribution (Supplementary Table S4). Both *Poxn* ^*ΔM22-B5*^ and *Poxn* ^*full-1*^ mutant flies differed from the wild type flies by showing a neutral response to the frequency of banana in the patch (see Fig. 2). The egg distribution of the *Poxn* ^*ΔM22-B5*^ mutant flies was affected by resource frequency (GLM: *χ* ^2^_1,495_ = 4.75, *P* = 0.03; Fig. 3), but only showed a marginally significant difference between the apple and banana substrates (GLM: *χ* ^2^_1,495_ = 3.78, *P* = 0.052), and no effect of the interaction between resource type and frequency (GLM: *χ* ^2^_1,495_ = 3.65, *P* = 0.06; Fig. 3). As this outcome did not conclusively indicate that the selection between the two resources was affected by the patch arrangements, we removed the non-significant interaction from the analysis and found no effects of resource type (GLM: *χ* ^2^_1,495_ = 1.56, *P* = 0.21) or resource frequency (GLM: *χ* ^2^_1,495_ = 0.44, *P* = 0.51), indicating random selection between the two oviposition resources. For the *Poxn* ^*full-1*^ flies, on the other hand, we found relatively similar egg distributions compared to that of the wild type flies on apple (Fig. 3). More specifically, we found an effect of resource type (GLM: *χ* ^2^_1,495_ = 5.41, *P* = 0.02), a strong increase in oviposition with resource frequency (GLM: *χ* ^2^_1,495_ = 30.63, *P* < 0.001), and an effect of the interaction between resource type and frequency (GLM: *χ* ^2^_1,495_ = 11.58, *P* < 0.001; Supplementary Table S3).

When comparing *w* ^1118^ with *Poxn* ^*SuperA-brainless107*^ or *Poxn* ^*SuperA158-119*^ respectively, there were no effects of any interactions with strain or of strain on its own (Supplementary Table S6), suggesting that the reconstruction of the gustatory sensilla restored wild type oviposition behavior. The absence of interactions in these comparisons with *w* ^1118^ also exclude the possibility that neurological deficiencies caused by the *white* or *Poxn* mutation had a direct effect on the oviposition behavior of the mutant flies included in our experiments (see Supplementary Table S1). Although the *mini-white* transgene partially restores *white*, unforeseen changes in the synthesis of dopamine and serotonin could have affected spatial memory and decision making by the mutants^42–44^. The absence of interactions between the strains suggests that the patterns found in our oviposition assay can be interpreted as effects of the actual sensory deficiencies rather than consequences of the genetic background in which the strains were constructed. Moreover, this hypothesis was also confirmed by the comparison between all gustatory mutants which indicated significant effects of interactions between resource type, resource frequency and strain on egg distribution (Supplementary Table S2). As expected, these interactions occurred due to apparent differences in the oviposition behavior of *Poxn* ^*ΔM22-B5*^ and *Poxn* ^*full-1*^ in comparison to that of *Poxn* ^*SuperA-brainless107*^ and *Poxn* ^*SuperA158-119*^ (Fig. 3; Supplementary Table S6). We can conclude from these results that the oviposition behavior of fruit flies is strongly governed by gustatory input. Entirely or partially losing the ability to evaluate direct contact non-volatile chemical cues resulted in oviposition patterns that deviated from the patterns we observed for wild type flies. This strongly suggests that gustatory mediated resource selection serves as an underlying behavioral mechanism for the occurrence of associational effects between resources for these flies.

## Discussion

Our results show that the occurrence of associational effects during oviposition by *Drosophila melanogaster* mainly relies on the use of non-volatile chemical cues, and less on the use of volatile cues. Our findings contrast with the main hypothesis that associational effects only occur through the misinterpretation of resource patch quality when selecting resources based upon long-range sensory information. These misinterpretations have been suggested to result from constraints in the capacity to evaluate resource availability from a distance, and therefore affect the likelihood of a female selecting a specific oviposition resource when arriving in a patch^11–13^. Partly due to the intrinsic limitations of testing this hypothesis under natural conditions, it has remained an obstacle for ecologists to empirically demonstrate whether associational effects actually depend on the combination of long- and short-range behavioral processes^25, 45^. To our knowledge, our study provides the first evidence that search behavior processes occurring at different spatial scales are not necessary in generating associational effects. Our results instead show that these effects can also be generated through sensory preferences solely based on short-range behavioral processes. Moreover, we found that deficiencies of distinct sensory modalities affect oviposition behavior in heterogeneous environments differently, and that such deficiencies either modulate the strength of associational effects between the two resources or eliminate the possibility of associational effects to occur.

We found that wild type fruit flies showed an increasing relative oviposition rate on apple substrates in patches with a higher frequency of banana, and a decreasing relative oviposition rate on banana substrates in patches with an increasing frequency of apple. This behavioral pattern can be translated into associational susceptibility (AS) for apple and associational resistance (AR) for banana. We also found that a complete functional loss of the gustatory sensilla in the *Poxn* ^*ΔM22-B5*^ flies entirely eliminated the patterns of the associational effects we observed for wild type fruit flies. We believe that the loss of associational effects for the *Poxn* ^*ΔM22-B5*^ flies was because female flies lacking the gustatory sensilla were unable to accurately assess the different substrates before making an oviposition choice, resulting in random resource selection^46–48^. As the *Poxn* ^*ΔM22-B5*^ flies retain the ability to select resources through olfactory cues, our results suggest that the observed associational effects are generated through the use of non-volatile chemical cues. Various studies show that freshly mated *D. melanogaster* females, as in our study, assess their oviposition environment by probing the available substrates with the proboscis and ovipositor before making any oviposition choice^37, 49, 50^. This behavior was also observed in our studies, and the inability of *Poxn* ^*ΔM22-B5*^ flies to use these sensory cues likely affected the relative egg-laying on the apple and banana substrates.

We also found that the partial olfactory deficiencies of the *Orco* ^2^ flies and the partial gustatory deficiencies of the *Poxn* ^*full-1*^ flies modulated the associational effects (Fig. 3), but the effects were still largely retained, in contrast to the *Poxn* ^*ΔM22-B5*^ flies. Moreover, neither the deficiencies in the acid-sensing (*IR8a* ^1^) nor in the amine-sensing pathways (*IR25a* ^2^) were able to alter the associational effects observed for wild type flies. Silbering *et al*.^51^ showed that *Orco* ^2^ flies have a stronger aversion to carboxylic acids (*IR8a* ligands), and an increased attraction to amines (*IR25a* ligands) in comparison to wild type flies, which could have influenced their ability to evaluate the resources and thereby affect the associational effects. Similarly, changes in the integration of sensory information by the *Poxn* ^*full-1*^ flies, which lack gustatory sensilla on the labellum, could have modulated the strength of associational effects. Gustatory sensilla on the labellum have been shown to be of high importance for substrate selection^37, 52–54^, making it likely that the absence of the integration of this sensory information during oviposition modulated the strength of associational effects. Interestingly, these results support our hypothesis of the importance of the ability to discriminate between resources as a mechanism underlying associational effects, especially as the rescue of the gustatory sensilla in the *Poxn* ^*SuperA-brainless107*^ and *Poxn* ^*SuperA158-119*^ flies successfully restored resource preference and associational effects (Fig. 3).

Our current study, showing that associational effects are generated through distinctive selection behavior by the consumer insect, rather than by a sensory misinterpretation as traditionally hypothesized^11–13^, may have implications for our general understanding of associational effects under natural conditions. Various studies have indicated the difficulty of reaching a consensus on the importance of search behavior by generalist versus specialist insects in the occurrence of associational effects^55–57^. Our results indicate that we should rather focus on the specific relationships between consumer- and resource traits to further elucidate the mechanisms generating associational effects^25, 45^. The applicability of our methods can be illustrated by a recent study, which suggested that the invasive agricultural pest species *Drosophila suzukii* integrates olfactory and gustatory sensory information differently during oviposition than other closely related species^58^. Hence, the applicability of our methods could lie in improving our understanding of the importance of sensory modalities in insect–host interactions in heterogeneous environments, and help to exploit insect host search behavior in future biological control strategies of pest species.

## Material and Methods

### Fly husbandry and mating of the flies

All flies were reared under controlled conditions (25 °C, 50% RH, 12:12 Light:Dark) in 28.5 × 95 mm rearing vials on a standard Bloomington diet containing corn syrup (115 mL/L), yeast (26 g/L), soy flour (15 g/L), cornmeal (110 g/L), agar (8.5 g/L) and propionic acid (7 ml/L). We anesthetized newly eclosed flies with CO_2_ and transferred them separated by sex into 28.5 × 95 mm rearing vials containing the standard diet, where we let them mature for 72 hours. Approximately two hours prior to the behavioral experiments we transferred one female and three males in rearing vials to allow for mating. The vials were checked every five minutes and only those females that had copulated for a minimum of 20 minutes were used in the oviposition experiments. We used Canton S, Dalby-HL and *w* ^1118^ to represent wild type oviposition behavior unaffected by sensory deficiencies. To determine the effects of olfactory deficiencies on oviposition behavior we used the *IR8a* ^1^ and *IR25a* ^2^ (both provided by Anders Enjin and Marcus Stensmyr) and *Orco* ^2^ mutant strains. Finally, we used *Poxn* ^*ΔM22-B5*^, *Poxn* ^*full-1*^ (both provided by Werner Boll) to determine the effect of gustatory deficiencies on oviposition behavior (see Supplementary Table S1).

All sensory mutants used in our experiments contain the *white* (w) mutation in their genetic background (see Supplementary Table S1 - genetic background), which codes for a transmembrane ABC transporter required for the synthesis of eye color pigment and the synthesis of dopamine and serotonin^59, 60^. Both dopamine and serotonin have been shown to be involved in spatial memory and decision making in complex environments^42–44^, and although the *white* mutation is partially restored by the insertion of the *mini-white* transgene marker in these strains, they most likely still have some neurological deficiencies caused by the *white* mutation^61, 62^. We used the *Poxn* ^*SuperA-brainless107*^ and *Poxn* ^*SuperA158-119*^ strains (both provided by Werner Boll), in which all gustatory sensilla are restored, as control strains for the behavior of the gustatory mutants and potential neurological deficiencies caused by *white* or *Poxn*. In both strains the *white* mutation is restored by the insertion of the *mini-white* trans-gene marker, but *Poxn* ^*SuperA-brainless107*^ still expresses the *Poxn* mutation in the central nervous system^39^, by comparing their behavior we effectively checked for potential effects of neurological deficiencies either caused by the *white* or *Poxn* mutation in the oviposition behavior of our flies.

### Oviposition experiments

Approximately 30 minutes after copulation we transferred individual females into polypropylene jars containing patches consisting of four oviposition discs placed in two rows separated by 8 mm. The oviposition discs were made of 18 mm filter paper discs (Grade 1003 – 12-15 μm pore size; Munktell Ahlstrom AB, Sweden) loaded with either apple or banana fruit pulp. All experiments were run with four oviposition discs to ensure that we could test the effects of patch heterogeneity along a sequence of different resource frequencies. We used the filter paper discs to ensure a standardized and accessible substrate for the females. The fruit substrates were made by pulverizing ripe apples (variety: Discovery, Sweden) and ripe bananas (Organic Cavendish – Dole, Dominican Republic) into a smooth fruit pulp. To preserve and standardize the quality of the fruit pulp we pulverized single batches of apple and banana, which were frozen in small quantities that could be easily defrosted prior to the experiments. The discs were loaded by slowly dragging the filter paper discs through the pulp, resulting in a thin and accessible layer of apple pulp (383.80 ± 8.69 mg) or banana pulp (331.12 ± 7.07 mg) on each disc. Over the entire time span of the experiments no fruit fluids leaked from the filter paper discs, creating resources with a distinct barrier which did not allow for any chemical diffusion found in similar setups made of agarose.

The jars were closed with airtight transparent lids and covered by an additional darkened lid to eliminate phototaxis of the flies to the top of the jar. We counted the number of eggs laid by Canton S, Dalby-HL, *w* ^1118^, *IR8a* ^1^ and *IR25a* ^2^ flies after 24 hours, but due to low egg laying during the first 24 hours we counted eggs of *Orco* ^2^, *Poxn* ^*ΔM22-B5*^, *Poxn* ^*full-1*^, *Poxn* ^*SuperA-brainless107*^ and *Poxn* ^*SuperA158-119*^ flies after 48 hours. Each treatment was replicated 25 times and consisted of one of the following patch arrangements; (1) four apple discs; (2) three apple and one banana disc; (3) two apple and two banana discs; (4) one apple and three banana discs; (5) four banana discs. We performed control trials in which Dalby-HL females were only offered a moistened filter paper disc to determine whether the flies would lay eggs on the filter paper discs within 24 hours regardless of the availability of fruit substrates. These tests showed that the females largely restrained themselves from laying eggs on moistened filter paper discs, suggesting that the discs without fruit pulp are not a suitable oviposition substrate. All experiments were conducted under the same controlled conditions as those under which the fly husbandry was maintained (25 °C, 50% RH, 12:12 Light:Dark).

### Statistical analysis

We analyzed the egg distribution along the different oviposition disc using Generalized Linear Mixed-effects Models (GLMM) with a negative binomial error distribution and the individual fly as a random factor using the glmmADMB package. We used negative binomial error distributions in our models based upon inspection of the normality of the residuals and the Q-Q plots, and the estimations of over-dispersion of initial models. In this analysis, each individual fly was represented by four separate values accounting for the number of eggs laid on each separate oviposition disc within the oviposition jar. We included the random factor to estimate relative, rather than absolute, oviposition rates by correcting for the number of oviposition discs per resource type in the patch. Consequently, this analysis compares the oviposition rates among single oviposition discs and identifies potential associational effects between individual resources^23^. The analysis for the individual strains included resource frequency, resource type and an interaction between these two factors as explanatory factors. In each analysis resource frequency was included as a continuous variable accounting for the proportion of banana oviposition discs in the patch, corresponding to values of 0, 0.25, 0.50, 0.75 or 1.

As all sensory mutants contain the *white* (*w*) mutation in their genetic background we decided that *w* ^1118^ would be the preferred strain to represent the wild type behavior in pairwise comparisons. All analysis that compared the behavior among strains included an additional explanatory factor to account for the strain, and all appropriate two-way interactions and a three-way interaction between strain, resource frequency and resource type. The models were selected using the car package^63^ for likelihood ratio tests based on *χ* ^2^ and Akaike’s information criterion in a step-wise backward selection process. The ggplot2 package was used to visualize the oviposition patterns within the different patch arrangements^64^. All analyses were carried out in R (v. 3.3.2; R Foundation for Statistical Computing, Vienna, AT).

### Data accessibility

The datasets generated and analyzed during the current study are available from the corresponding author upon reasonable request.

## Electronic supplementary material

Supporting Information
