# Supplementary material for: Sensory mutations in Drosophila melanogaster influence associational effects between resources during oviposition
Source: Sci Rep. 2017 Aug 24;7:9352. doi: 10.1038/s41598-017-09728-7 (PMC5570953; doi:10.1038/s41598-017-09728-7)
Supplement: Supplementary file 1 — Supporting Information [file 41598_2017_9728_MOESM1_ESM.pdf]

Sensory mutations in *Drosophila melanogaster* influence associational effects between resources during oviposition

Supplementary Information

Author affiliation:

Thomas A. Verschut (http://orcid.org/0000-0003-0130-6485)
Department of Ecology, Environment and Plant Sciences, Stockholm University, 106 91 Stockholm, Sweden.

Mikael A. Carlsson (http://orcid.org/0000-0002-9190-6873)
Department of Zoology, Stockholm University, 106 91 Stockholm, Sweden.

Peter Anderson (http://orcid.org/0000-0003-4105-8236)
Department of Plant Protection Biology, Swedish University of Agricultural Sciences, Box 102, 230 53 Alnarp, Sweden.

Peter A. Hambäck (http://orcid.org/0000-0001-6362-6199)
Department of Ecology, Environment and Plant Sciences, Stockholm University, 106 91 Stockholm, Sweden.

Corresponding author:
Thomas A. Verschut
Department of Ecology, Environment and Plant Sciences, Stockholm University, 106 91 Stockholm, Sweden. Email: thomas.verschut@su.se Phone: +46(0)8 16 38 49

Supplementary information

Table S1. Summary of the strains used in the oviposition assays. References to additional background information is given when applicable and the Bloomington Drosophila Stock Centre strain number is given between round brackets when available.

	Strain	Genetic Background	Description	Reference
**Wild Type**	Canton S	-	Commonly used wild type strain.	(64349)
	Dalby-HL	-	Recently established wild type strain originating from Dalby, Sweden.	[1]
	w^{1118}	-	All mutants used in this study are constructed in a *white* (*w*) background. This gene codes for a transmembrane transporter involved in the uptake of guanine and tryptophan. Besides impaired vision, due to the lack of optical insulation, these flies have lowered levels of dopamine and serotonin neurotransmitters.	[2, 3] (6326)
**Olfactory mutants**	$Orco^2$	*w**; TI(TI)$Orco^2$	$Orco^2$ flies lack the olfactory coreceptor (Orco) normally operating in the receptor complex with all other odorant receptors (OR). Due to this mutation $Orco^2$ flies have no functioning odorant receptor complex and cannot perceive any odors normally detected by ORs located in the maxillary palps and third antennal segment. Several odorants are still detected by the ionotropic receptors (IR).	[4] (23130)
	$IR8a^1$	*w** TI(TI)$Ir8a^1$; Bl1 L2/CyO	$IR8a^1$ flies lack the dedicated ionotropic coreceptor *IR8a* involved in pathways of acid-sensitive IRs in the sacculus and the third segment of the antenna. Due to the mutation $IR8a^1$ flies have a reduced ability to sense carboxylic acids.	[5-7] (41744)
	$IR25a^2$	*w**; TI(TI)$Ir25a^2$/CyO	$IR25a^2$ flies lack the universal ionotropic coreceptor *IR25a* that involved in pathways of amine-sensitive IRs in the labellum and the third segment of the antenna. Due to the mutation $IR25a^2$ flies have a reduced ability to sense amines.	[5-7] (41737)

Continuation of Table S1. Summary of the strains used in the oviposition assays. References to additional background information is given when applicable and the Bloomington Drosophila Stock Center strain number is given between round brackets when available.

	Strain	Genetic Background	Description	Reference
**Gustatory mutants**	*Poxn*$^{\Delta M22\text{-}B5}$	w^{1118}; $\mathrm{Poxn}^{\Delta M22\text{-}B5}$; $\mathrm{P}^{(W6\ Poxn\_resc)\Delta SfoBs127}$	The *Pox-neuro* (*Poxn)* mutation turns all poly-innervated gustatory bristles into mono-innervated mechanosensory bristles. In *Poxn*$^{\Delta M22\text{-}B5}$ none of the gustatory bristles function, but the functioning of the central nervous system is partly rescued, and the genitalia and the segmentation of the antennae and legs is completely rescued by the $\mathrm{P}^{(W6\ Poxn\_resc)\Delta SfoBs127}$ transgene.	[8-10]
	Poxn$^{full\text{-}1}$	w^{1118}; $\mathrm{Poxn}^{\Delta M22\text{-}B5}$; $\mathrm{P}^{(W6\ Poxn\_resc)full\text{-}1}$	The *full-1* transgene rescues the function of the gustatory bristles on the legs and anterior wing margins, but does not restore the gustatory bristles on the labellum of the *Poxn* flies. The *full-1* transgene also rescues all deficiencies in the *Poxn* central nervous system.	[8]
	Poxn$^{SuperA\text{-}brainless107}$	w^{1118}; $\mathrm{Poxn}^{\Delta M22\text{-}B5}$; $\mathrm{P}^{(W6\ Poxn\_resc)SuperA\text{-}brainless107}$	The *SuperA-brainless107* trans-gene restores the gustatory bristles in *Poxn* flies but does not restore the deficiencies in the *Poxn* central nervous system.	[8, 11]
	Poxn$^{SuperA158\text{-}119}$	w^{1118}; $\mathrm{Poxn}^{\Delta M22\text{-}B5}$; $\mathrm{P}^{(W6\ Poxn\_resc)SuperA158\text{-}119}$	The *SuperA158-119* transgene restores the gustatory bristles and the deficiencies in the *Poxn* central nervous system.	[8, 11]

[1] Ruebenbauer *et al.* (2008) *Curr. Biol.* **18**, 1438-1443; [2] Sitaraman *et al.* (2008) *Proc. Natl. Acad. Sci. USA* **105**, 5579-5584; [3] Borycz *et al.* (2008) *J. Exp. Biol.* **211**, 3454-3466; [4] Larsson *et al.* (2004) *Neuron* **43**, 703-714; [5] Abuin *et al.* (2011) *Neuron* **69**, 44-60.; [6] Benton *et al.* (2009) *Cell* **136**, 149-162.; [7] Silbering & Benton (2010) *EMBO Rep.* **11**, 173-179; [8] Boll & Noll (2002) *Development* **129**, 5667-5681; [9] Awasaki & Kimura (1997) *J. Neurobiol.* **32**, 707-721; [10] Awasaki & Kimura (2001) *Dev. Genes Evol.* **211**, 20-29; [11] Minocha *et al.* (2017) *PLOS ONE* **12**, e0176002.

Supplementary results

Table S2. Summary of likelihood ratio tests (χ^2) of the relative oviposition patterns of the different sensory types of *Drosophila melanogaster.* Explanations of each strain belonging to the different sensory types can be found in Table S1. Resource accounts for the type of fruit pulp loaded on the oviposition discs (i.e. apple or banana), and frequency accounts for the proportion of banana oviposition discs in the oviposition assay. Only those interactions relevant for the behavioral patterns or left after backwards selection are provided in the table.

**Comparison**	**Factors**	χ^2	***df***	***P***
Wild type	Strain	3.91	2, 1489	0.14
	Frequency	78.84	1, 1489	**<0.001**
	Resource	61.64	1, 1489	**<0.001**
	Strain *x* Frequency	0.27	2, 1489	0.86
	Strain *x* Resource	2.12	2, 1489	0.34
	Frequency *x* Resource	11.35	1, 1489	**<0.001**
Olfactory mutants	Strain	5.56	2, 1487	0.06
	Frequency	41.51	1, 1487	**<0.001**
	Resource	58.18	1, 1487	**<0.001**
	Strain *x* Frequency	3.56	2, 1487	0.17
	Strain *x* Resource	1.39	2, 1487	0.50
	Frequency *x* Resource	0.19	1, 1487	0.66
	Strain *x* Frequency *x* Resource	8.81	2, 1487	**0.01**
Gustatory mutants	Strain	21.36	3, 1986	**<0.001**
	Frequency	6.04	1, 1986	**0.01**
	Resource	7.45	1, 1986	**0.006**
	Strain *x* Frequency	73.05	3, 1986	**<0.001**
	Strain *x* Resource	264.15	3, 1986	**<0.001**
	Frequency *x* Resource	7.46	1, 1986	**0.006**

Table S3. Summary of likelihood ratio tests (χ^2) of the relative oviposition patterns of the individual strains. Resource accounts for the type of fruit pulp loaded on the oviposition discs (i.e. apple or banana), and frequency accounts for the proportion of banana oviposition discs in the oviposition assay. Only those interactions relevant for the behavioral patterns or left after backwards selection are provided in the table.

	Strain	Factors	χ^2	*df*	*P*
**Wild Type**	Canton-S	Frequency	45.82	1, 495	**<0.001**
		Resource	20.08	1, 495	**<0.001**
		Frequency *x* Resource	5.67	1, 495	**0.02**
	Dalby-HL	Frequency	142.03	1, 495	**<0.001**
		Resource	71.56	1, 495	**<0.001**
		Frequency *x* Resource	4.84	1, 495	**0.03**
	w^{1118}	Frequency	33.17	1, 495	**<0.001**
		Resource	27.08	1, 495	**<0.001**
		Frequency *x* Resource	1.69	1, 495	0.19
**Olfactory mutants**	$IR8a^{1}$	Frequency	43.72	1, 495	**<0.001**
		Resource	59.93	1, 495	**<0.001**
		Frequency *x* Resource	0.32	1, 495	0.57
	$IR25a^{2}$	Frequency	27.98	1, 495	**<0.001**
		Resource	44.14	1, 495	**<0.001**
		Frequency *x* Resource	0.06	1, 495	0.79
	$Orco^{2}$	Frequency	24.77	1, 495	**<0.001**
		Resource	74.69	1, 495	**<0.001**
		Frequency *x* Resource	23.77	1, 495	**<0.001**
**Gustatory mutants**	$Poxn^{\Delta M22\text{-}B5}$	Frequency	4.75	1, 495	**0.03**
		Resource	3.78	1, 495	0.052
		Frequency *x* Resource	3.65	1, 495	0.06
	$Poxn^{full\text{-}1}$	Frequency	30.63	1, 495	**<0.001**
		Resource	5.41	1, 495	**0.02**
		Frequency *x* Resource	11.58	1, 495	**<0.001**
	$Poxn^{SuperA\text{-}brainless107}$	Frequency	58.32	1, 495	**<0.001**
		Resource	68.25	1, 495	**<0.001**
		Frequency *x* Resource	0.34	1, 495	0.56
	$Poxn^{SuperA158\text{-}119}$	Frequency	51.39	1, 495	**<0.001**
		Resource	42.18	1, 495	**<0.001**
		Frequency *x* Resource	0.27	1, 495	0.61

Table S4. Summary of likelihood ratio tests (χ^2) of the planned comparisons between w^{1118} and the sensory mutants. Resource accounts for the type of fruit pulp loaded on the oviposition discs (i.e. apple or banana), and frequency accounts for the proportion of banana oviposition discs in the oviposition assay. Only those interactions relevant for the behavioral patterns or left after backwards selection are provided in the table.

**Comparison**	**Factors**	χ^2	***df***	***P***
w^{1118} *x Orco*^{2}	Strain	0.01	1, 990	0.99
	Frequency	16.56	1, 990	**<0.001**
	Resource	56.88	1, 990	**<0.001**
	Strain *x* Frequency	3.22	1, 990	0.07
	Strain *x* Resource	5.65	1, 990	**0.02**
	Frequency *x* Resource	12.71	1, 990	**<0.001**
	Strain *x* Frequency *x* Resource	16.45	1, 990	**<0.001**
w^{1118} *x IR8a*^{1}	Strain	0.62	1, 991	0.43
	Frequency	71.02	1, 991	**<0.001**
	Resource	80.26	1, 991	**<0.001**
	Strain *x* Frequency	6.03	1, 991	**0.01**
	Strain *x* Resource	5.97	1, 991	**0.01**
	Frequency *x* Resource	0.26	1, 991	0.61
w^{1118} *x IR25a*^{2}	Strain	2.85	1, 991	0.09
	Frequency	48.12	1, 991	**<0.001**
	Resource	67.18	1, 991	**<0.001**
	Strain *x* Frequency	1.25	1, 991	0.26
	Strain *x* Resource	3.68	1, 991	0.055
	Frequency *x* Resource	1.05	1, 991	0.31
w^{1118} *x Poxn*$^{\Delta M22\text{-}B5}$	Strain	10.35	1, 991	**0.001**
	Frequency	4.99	1, 991	**0.03**
	Resource	3.69	1, 991	**0.05**
	Strain *x* Frequency	19.15	1, 991	**<0.001**
	Strain *x* Resource	85.18	1, 991	**<0.001**
	Frequency *x* Resource	4.59	1, 991	**0.03**
w^{1118} *x Poxn*$^{full\text{-}1}$	Strain	0.97	1, 991	0.32
	Frequency	27.60	1, 991	**<0.001**
	Resource	12.18	1, 991	**<0.001**
	Strain *x* Frequency	4.11	1, 991	**0.04**
	Strain *x* Resource	6.75	1, 991	**0.009**
	Frequency *x* Resource	10.53	1, 991	**0.001**
w^{1118} *x Poxn*$^{SuperA\text{-}brainless107}$	Strain	0.09	1, 991	0.76
	Frequency	64.59	1, 991	**<0.001**
	Resource	74.74	1, 991	**<0.001**
	Strain *x* Frequency	1.29	1, 991	0.26
	Strain *x* Resource	0.89	1, 991	0.35
	Frequency *x* Resource	0.50	1, 991	0.48
w^{1118} *x Poxn*$^{SuperA158\text{-}119}$	Strain	2.75	1, 991	0.09
	Frequency	62.84	1, 991	**<0.001**
	Resource	55.99	1, 991	**<0.001**
	Strain *x* Frequency	1.85	1, 991	0.17
	Strain *x* Resource	0.57	1, 991	0.45
	Frequency *x* Resource	1.79	1, 991	0.18

Table S5. Summary of likelihood ratio tests (χ^2) of the planned comparisons between the olfactory mutants. Resource accounts for the type of fruit pulp loaded on the oviposition discs (i.e. apple or banana), and frequency accounts for the proportion of banana oviposition discs in the oviposition assay. Only those interactions relevant for the behavioral patterns or left after backwards selection are provided in the table.

**Comparison**	**Factors**	χ^2	***df***	***P***
Orco² x *IR8a¹*	Strain	3.49	1, 990	0.06
	Frequency	64.59	1, 990	**<0.001**
	Resource	78.53	1, 990	**<0.001**
	Strain *x* Frequency	7.14	1, 990	0.007
	Strain *x* Resource	0.01	1, 990	0.95
	Frequency *x* Resource	0.39	1, 990	0.53
	Strain *x* Frequency *x* Resource	5.75	1, 990	**0.02**
Orco² x *IR25a²*	Strain	5.37	1, 990	**0.02**
	Frequency	45.07	1, 990	**<0.001**
	Resource	61.54	1, 990	**<0.001**
	Strain *x* Frequency	3.49	1, 990	0.06
	Strain *x* Resource	0.62	1, 990	0.43
	Frequency *x* Resource	0.26	1, 990	0.61
	Strain *x* Frequency *x* Resource	8.54	1, 990	**0.003**
IR8a¹ x *IR25a²*	Strain	0.932	1, 991	0.33
	Frequency	37.08	1, 991	**<0.001**
	Resource	76.29	1, 991	**<0.001**
	Strain *x* Frequency	1.57	1, 991	0.21
	Strain *x* Resource	0.21	1, 991	0.65
	Frequency *x* Resource	0.03	1, 991	0.86

Table S6. Summary of likelihood ratio tests (χ^2) of the planned comparisons between the gustatory mutants. Resource accounts for the type of fruit pulp loaded on the oviposition discs (i.e. apple or banana), and frequency accounts for the proportion of banana oviposition discs in the oviposition assay. Only those interactions relevant for the behavioral patterns or left after backwards selection are provided in the table.

**Comparison**	**Factors**	χ^2	*df*	***P***
Poxn$^{\Delta M22\text{-}B5}$ *x Poxn*$^{full\text{-}1}$	Strain	4.79	1, 991	**0.03**
	Frequency	9.94	1, 991	**0.002**
	Resource	7.72	1, 991	**0.005**
	Strain *x* Frequency	5.30	1, 991	**0.02**
	Strain *x* Resource	47.28	1, 991	**<0.001**
	Frequency *x* Resource	15.02	1, 991	**<0.001**
Poxn$^{\Delta M22\text{-}B5}$ *x Poxn*$^{SuperA\text{-}brainless107}$	Strain	18.59	1, 991	**<0.001**
	Frequency	3.14	1, 991	0.08
	Resource	2.70	1, 991	0.10
	Strain *x* Frequency	40.08	1, 991	**<0.001**
	Strain *x* Resource	131.34	1, 991	**<0.001**
	Frequency *x* Resource	0.78	1, 991	0.38
Poxn$^{\Delta M22\text{-}B5}$ *x Poxn*$^{SuperA158\text{-}119}$	Strain	3.31	1, 991	0.07
	Frequency	3.43	1, 991	0.06
	Resource	4.73	1, 991	**0.03**
	Strain *x* Frequency	37.59	1, 991	**<0.001**
	Strain *x* Resource	108.63	1, 991	**<0.001**
	Frequency *x* Resource	2.45	1, 991	0.11
Poxn$^{full\text{-}1}$ *x Poxn*$^{SuperA\text{-}brainless107}$	Strain	8.91	1, 990	**0.003**
	Frequency	51.02	1, 990	**<0.001**
	Resource	53.91	1, 990	**<0.001**
	Strain *x* Frequency	0.48	1, 990	0.49
	Strain *x* Resource	15.71	1, 990	**<0.001**
	Frequency *x* Resource	0.11	1, 990	0.74
	Strain *x* Frequency *x* Resource	7.99	1, 990	**0.005**
Poxn$^{full\text{-}1}$ *x Poxn*$^{SuperA158\text{-}119}$	Strain	0.43	1, 991	0.51
	Frequency	85.15	1, 991	**<0.001**
	Resource	45.77	1, 991	**<0.001**
	Strain *x* Frequency	12.31	1, 991	**<0.001**
	Strain *x* Resource	11.82	1, 991	**<0.001**
	Frequency *x* Resource	7.99	1, 991	**0.004**
Poxn$^{SuperA158\text{-}119}$ *x Poxn*$^{SuperA\text{-}brainless107}$	Strain	4.85	1, 991	**0.03**
	Frequency	70.91	1, 991	**<0.001**
	Resource	83.91	1, 991	**<0.001**
	Strain *x* Frequency	0.13	1, 991	0.71
	Strain *x* Resource	0.01	1, 991	0.92
	Frequency *x* Resource	0.01	1, 991	0.99
